# Genetic Differentiation and Spatial Structure of *Phellinus noxius*, the Causal Agent of Brown Root Rot of Woody Plants in Japan

**DOI:** 10.1371/journal.pone.0141792

**Published:** 2015-10-29

**Authors:** Mitsuteru Akiba, Yuko Ota, Isheng J. Tsai, Tsutomu Hattori, Norio Sahashi, Taisei Kikuchi

**Affiliations:** 1 Department of Forest Microbiology, Forestry and Forest Products Research Institute, Tsukuba, Japan; 2 Biodiversity Research Center, Academia Sinica, Taipei, Taiwan; 3 Division of Parasitology, Faculty of Medicine, University of Miyazaki, Miyazaki, Japan; Universita degli Studi di Pisa, ITALY

## Abstract

*Phellinus noxius* is a pathogenic fungus that causes brown root rot disease in a variety of tree species. This fungus is distributed in tropical and sub-tropical regions of Southeast and East Asia, Oceania, Australia, Central America and Africa. In Japan, it was first discovered on Ishigaki Island in Okinawa Prefecture in 1988; since then, it has been found on several of the Ryukyu Islands. Recently, this fungus was identified from the Ogasawara (Bonin) Islands, where it has killed trees, including rare endemic tree species. For effective control or quarantine methods, it is important to clarify whether the Japanese populations of *P*. *noxius* are indigenous to the area or if they have been introduced from other areas. We developed 20 microsatellite markers from genome assembly of *P*. *noxius* and genotyped 128 isolates from 12 of the Ryukyu Islands and 3 of the Ogasawara Islands. All isolates had unique genotypes, indicating that basidiospore infection is a primary dissemination method for the formation of new disease foci. Genetic structure analyses strongly supported genetic differentiation between the Ryukyu populations and the Ogasawara populations of *P*. *noxius*. High polymorphism of microsatellite loci suggests that Japanese populations are indigenous or were introduced a very long time ago. We discuss differences in invasion patterns between the Ryukyu Islands and the Ogasawara Islands.

## Introduction

*Phellinus noxius* (Corner) G. Cunn. (Hymenochaetaceae) is a pathogenic fungus that causes brown root rot disease in a variety of tree species [1–7]. The fungus is distributed in tropical and sub-tropical regions in Southeast and East Asia, Oceania, Australia, Central America and Africa [6,8–14]. Infection causes slow and reduced growth in trees, discolouration and wilting of leaves, defoliation, and dieback of branches [14,15]. Most affected trees eventually die, and in some cases, the fungus causes the rapid wilt and death of the tree within a few months of infection [8,11,12]. The host range of the fungus is very wide [1,6], showing little host specificity [5,7,16], and to date more than 200 woody plant species representing 59 families have been recorded as host plants[12]. The life cycle of *P*. *noxius* is similar to that of other important forest pathogens, such as *Phellinus sulphurascens* Pilát that causes laminated root rot of conifers [17] and *Armillaria* spp. that cause Armillaria root rot of woody plants [18]. The fungus infects host trees via root-to-root contact from adjacent infected trees, or from wood debris of dead trees where *P*. *noxius* can persist saprophytically more than ten years [19]. Basidiospores may function to establish new disease foci [1,14], but remain undocumented in *P*. *noxius*.

In Japan, brown root rot was first found in windbreaks composed of *Casuarina equisetifolia* L. on Ishigaki Island in Okinawa Prefecture in 1988 [20]. Since then, the disease has gained increasing attention as it has appeared on several islands of the Ryukyu Islands in both Okinawa and Kagoshima Prefectures, causing serious problems for shade, windbreak, and ornamental or landscape trees [14,21–23]. Amami-Oshima Island currently represents the northernmost distribution point of the disease [23]. In 2012, this fungus was identified on the Ogasawara (Bonin) Islands, oceanic islands located approximately 1,000 km south of Tokyo, where it killed trees, including rare species endemic to the islands (Sahashi et al. personal communication).

*Phellinus noxius* is suspected to be indigenous to many tropical or subtropical areas throughout the world [8]; however, whether *P*. *noxius* in Japan is indigenous or has been introduced from other areas remains unclear. *Phellinus noxius* on the Ogasawara Islands was possibly introduced from the Ryukyu Islands, as several tree species, including *Bischofia javanica* Blume and *Pinus luchuensis* Mayr were introduced to these islands from the Ryukyus as timber or fuel trees in the early 1900s [24]. To establish effective control or quarantine methods for brown root rot, it is important to first determine whether the Japanese populations are indigenous to the area or introduced from other areas.

Simple sequence repeats (SSRs) or microsatellites are a group of DNA sequences with repeating units of 2–6 base pairs (bp) that are abundant in most genomes exhibiting high levels of polymorphism [25,26]. Hence, SSRs are useful molecular markers for analysing genetic diversity and have recently been used for a robust assessment of population structure in various plant pathogens [27–30]. In this study, we developed microsatellite markers based on the *de novo* sequencing assembly of a Japanese isolate and then analysed genetic diversity or genetic structure in *P*. *noxius* in Japan. Although population genetics studies using microsatellite markers have been conducted for other similar root rot pathogenic fungi distributed in cool- or warm-temperate areas, including *Armillaria* spp. [31–33] and *Heterobasidion* spp. [29,34], this study is the first to examine the population genetics of wood-decay and tree pathogenic fungi in a tropical or subtropical area.

## Materials and Methods

### Isolates

**Fig 1:**
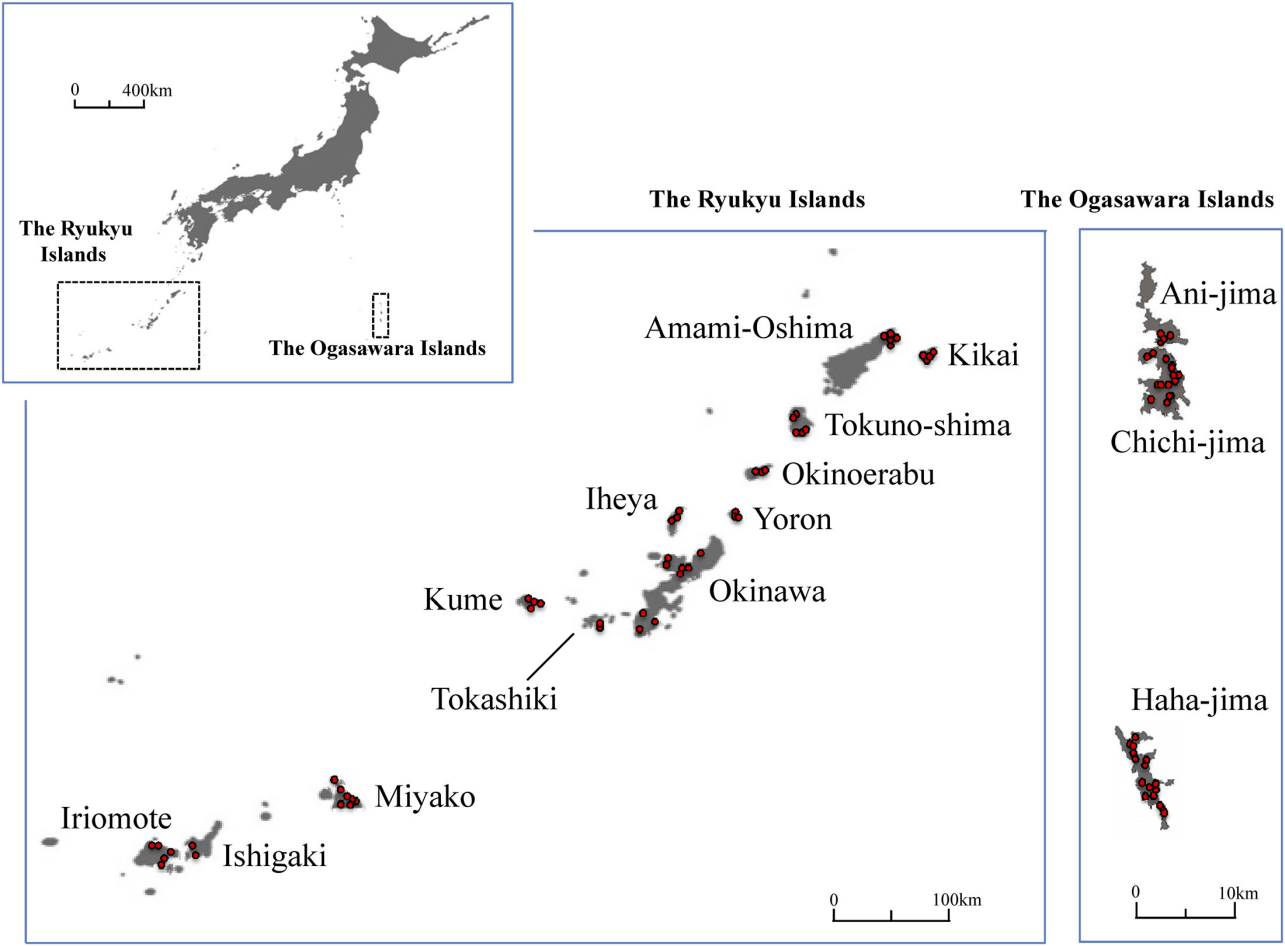
Location of the sampling sites for *P*. *noxius* isolates used in this study. Red circles indicate the sampling sites for each isolate.

The isolates of *Phellinus noxius* used in this study are listed in Table 1. We collected infected root samples or basidiocarps (for isolate KPN246 only) from 12 of the Ryukyu Islands in 1990–2010 [14] and from 3 of the Ogasawara Islands in 2012–2013 (Fig 1). *Phellinus noxius* was isolated using the methods described in Sahashi et al. (2012) [14]. Isolates were cultured on potato dextrose agar (PDA; Nissui, Tokyo, Japan) in test tubes and were maintained at 25°C using periodical subculture at the Forestry and Forest Products Research Institute (FFPRI, Tsukuba, Japan). Unless two isolates from the same region were found genetically incompatible, one isolate per disease foci was used for subsequent analysis. Moreover, one isolate (P919-02W.1) of *P*. *noxius* from Pohnpei Island, Federated Stated of Micronesia, isolated by Y. Ota and N. Sahashi in 2013 was included in the development of the microsatellite marker to guarantee the robustness of the markers for future worldwide analyses.

**Table 1:** Location, hosts, ploidy for *Phellinus noxius* isolates used in this study.

Isolate	Year	Prefecture, Country	Island	Latitude (°N)	Longitude (°E)	Host	Ploidy^a^
**KPN56**	2004	Kagoshima, Japan	Amami-Oshima	28.47552	129.70608	*Cinnamomum yabunikkei*	Diploid
**KPN21** ^b^	2002			28.47152	129.71311	*Amygdalus persica*	Haploid
**KPN57**	2004			28.47152	129.71311	*Pittosporum tobira*	Diploid
**KPN53**	2004			28.44750	129.67508	*Elaeocarpus zollingeri*	Haploid
**KPN19**	2002			28.43551	129.70814	*Glochidion obovatum*	Diploid
**KPN65**	2005			28.41099	129.66938	*Rhaphiolepis indica* var. *umbellata*	Haploid
**KPN24**	2003			28.41224	129.62836	*Litsea japonica*	Diploid
**KPN23**	2003			28.47518	129.60858	*Cinnamomum yabunikkei*	Diploid
**KPN59**	2006		Kikai	28.31838	129.92567	*Cinnamomum yabunikkei*	Diploid
**KPN92** ^b^	2007			28.31838	129.92567	*Casuarina equisetifolia*	Diploid
**KPN62**	2006			28.31017	129.98400	*Cinnamomum yabunikkei*	Diploid
**KPN63**	2006			28.34193	130.00869	*Cinnamomum yabunikkei*	Haploid
**KPN64**	2006			28.33032	129.99694	*Cinnamomum yabunikkei*	Haploid
**KPN98**	2007			28.30613	129.98222	*Cinnamomum yabunikkei*	Diploid
**KPN9**	2001			28.28914	129.96335	*Cinnamomum yabunikkei*	Haploid
**KPN84**	2007		Tokunoshima	27.83103	128.88351	*Rhaphiolepis indica* var. *umbellata*	Diploid
**KPN87**	2007			27.71696	128.89028	*Rhaphiolepis indica* var. *umbellata*	Diploid
**KPN90**	2007			27.69066	128.99754	*Cinnamomum yabunikkei*	Haploid
**KPN13**	2001			27.68011	128.97378	*Casuarina equisetifolia*	Diploid
**KPN1** ^b^	1999			27.68651	128.93357	*Ardisia sieboldii*	Haploid
**KPN7**	1999			27.84536	128.90158	*Nandina domestica*	Haploid
**KPN26**	2003		Okinoerabu	27.40112	128.65975	*Rhaphiolepis indica* var. *umbellata*	Diploid
**KPN28** ^c^	2003			27.39265	128.64336	*Ficus microcarpa*	Haploid
**KPN30**	2003			27.39265	128.64336	*Ficus virgata*	Haploid
**KPN31**	2003			27.38978	128.59201	*Elaeocarpus zollingeri*	Diploid
**KPN47**	2004		Yoron	27.06370	128.43158	*Litsea japonica*	Haploid
**KPN49**	2004			27.06370	128.43158	*Cinnamomum yabunikkei*	Haploid
**KPN46**	2004			27.06193	128.42653	*Cinnamomum yabunikkei*	Haploid
**KPN50**	2004			27.02536	128.45194	*Hibiscus rosa-sinensis*	Haploid
**KPN44**	2004			27.03909	128.42886	*Cinnamomum yabunikkei*	Haploid
**KPN15**	2001			unknown	unknown	*Ficus microcarpa*	Haploid
**KPN35**	2003	Okinawa, Japan	Okinawa	26.24259	127.68431	*Hibiscus tiliaceus*	Diploid
**KPN39**	2003			26.59401	127.96972	*Distylium racemosum*	Haploid
**KPN41**	2003			26.60516	127.99717	*Litchi chinensis*	Haploid
**KPN42**	2003			26.68154	127.88200	*Casuarina equisetifolia*	Diploid
**KPN43**	2003			26.11156	127.66983	*Casuarina equisetifolia*	Diploid
**KPN135**	2010			26.61851	127.98339	*Casuarina equisetifolia*	Diploid
**KPN141** ^c^	2010			26.62493	128.02231	*Casuarina equisetifolia*	Diploid
**KPN142**	2010			26.69022	128.11341	*Cinnamomum doederleinii*	Diploid
**KPN145** ^b^	2010			26.17121	127.78491	*Casuarina equisetifolia*	Diploid
**KPN147**	2010			26.17121	127.78491	*Casuarina equisetifolia*	Haploid
**KPN449**	2014			26.69177	127.87907	*Ficus microcarpa*	Diploid
**KPN169**	2012		Iheya	27.04023	127.97202	*Casuarina equisetifolia*	Diploid
**KPN172**	2012			27.08177	128.00682	*Casuarina equisetifolia*	Diploid
**KPN174**	2012			27.06320	127.97353	*Cinnamomum doederleinii*	Diploid
**KPN175**	2012			26.99897	127.92585	*Casuarina equisetifolia*	Diploid
**KPN178**	2012			27.02864	127.96016	*Machilus thunbergii*	Diploid
**KPN127**	2010		Kume	26.34267	126.81732	*Machilus thunbergii*	Diploid
**KPN128**	2010			26.35952	126.80051	*Cinnamomum doederleinii*	Diploid
**KPN129**	2010			26.36412	126.79836	*Cinnamomum yabunikkei*	Diploid
**KPN131**	2010			26.38016	126.78082	*Cerasus campanulata*	Diploid
**KPN132** ^c^	2010			26.31791	126.77623	*Casuarina equisetifolia*	Haploid
**KPN133**	2010			26.31691	126.77556	*Cinnamomum yabunikkei*	Diploid
**KPN161**	2012		Tokashiki	26.15993	127.35199	*Cinnamomum doederleinii*	Diploid
**KPN163**	2012			26.15922	127.35211	*Cinnamomum doederleinii*	Diploid
**KPN164**	2012			26.15922	127.35211	Broadleaf tree	Diploid
**KPN168**	2012			26.15518	127.34779	*Rhaphiolepis indica* var. *umbellata*	Diploid
**KPN101**	2009		Miyako	24.84565	125.29655	*Calophyllum inophyllum*	Diploid
**KPN104**	2009			24.86451	125.29092	*Acacia confusa*	Haploid
**KPN106**	2009			24.93732	125.23983	*Casuarina equisetifolia*	Diploid
**KPN110**	2009			24.82459	125.31910	*Casuarina equisetifolia*	Diploid
**KPN112**	2009			24.82365	125.31932	*Leucaena leucocephala*	Diploid
**KPN116**	2009			24.81788	125.31527	*Heliotropium foertherianum*	Diploid
**KPN117**	2009			24.80366	125.32783	*Ceiba speciosa*	Diploid
**KPN119**	2009			24.79894	125.31681	*Erythrina variegata*	Diploid
**KPN121** ^b^	2009			24.78544	125.35792	*Casuarina equisetifolia*	Haploid
**KPN122**	2009			24.77356	125.38921	*Casuarina equisetifolia*	Diploid
**KPN123**	2009			24.76292	125.39249	*Casuarina equisetifolia*	Diploid
**KPN124**	2009			24.73607	125.36335	*Casuarina equisetifolia*	Diploid
**KPN126**	2009			24.74007	125.30943	Broadleaf tree	Haploid
**KPN76**	2007		Ishigaki	24.37751	124.19691	*Eugenia uniflora*	Diploid
**KPN78**	2007			24.37654	124.19498	*Garcinia subelliptica*	Diploid
**KPN79**	2007			24.34543	124.15974	*Diospyros egbert-walkeri*	Diploid
**KPN80** ^c^	2007			24.34441	124.15791	*Ehretia philippinensis*	Haploid
**KPN82**	2007		Iriomote	24.27107	123.87912	*Melia azedarach*	Haploid
**KPN149**	2010			24.27181	123.87799	*Melia azedarach*	Diploid
**KPN152**	2010			24.42700	123.77603	*Casuarina equisetifolia*	Diploid
**KPN156**	2010			24.39858	123.77030	*Leucaena leucocephala*	Diploid
**KPN157** ^b^ ^**,**^ ^d^	2010			24.40160	123.77489	*Leucaena leucocephala*	Haploid
**KPN159**	2010			24.40160	123.77489	*Macaranga tanarius* var. *tomentosa*	Diploid
**KPN363**	2013			24.39606	123.80214	*Ceiba speciosa*	Diploid
**KPN362**	2013			24.29768	123.87156	*Morus australis*	Diploid
**KPN364**	2013			24.27054	123.84159	*Leucaena leucocephala*	Diploid
**KPN365**	2013			24.42606	123.79222	*Calophyllum inophyllum*	Diploid
**KPN259**	2013	Tokyo, Japan	Ani-jima	27.11748	142.20807	*Terminalia catappa*	Diploid
**KPN256** ^b^	2013			27.11709	142.20885	*Planchonella obovata*	Diploid
**KPN261**	2013			27.11665	142.21327	*Distylium lepidotum*	Diploid
**KPN264**	2013			27.11064	142.20714	*Rhaphiolepis indica* var. *umbellata*	Diploid
**KPN179**	2012		Chichi-jima	27.07750	142.21767	*Neolitsea sericea* var. *aurata*	Diploid
**KPN180**	2012			27.08063	142.22117	*Rhaphiolepis indica* var. *umbellata*	Diploid
**KPN186**	2012			27.08093	142.22260	*Neolitsea sericea* var. *aurata*	Diploid
**KPN190**	2012			27.07420	142.22237	*Planchonella obovata*	Diploid
**KPN294**	2013			27.08575	142.21750	*Casuarina equisetifolia*	Diploid
**KPN194**	2012			27.05400	142.20829	*Trema orientalis*	Diploid
**KPN200**	2012			27.05117	142.21042	*Ardisia sieboldii*	Diploid
**KPN247**	2012			27.06806	142.20616	*Ficus bengalensis*	Diploid
**KPN280** ^c^	2013			27.08678	142.21696	*Ardisia sieboldii*	Diploid
**KPN255**	2012			27.09332	142.18943	*Leucaena leucocephala*	Diploid
**KPN267**	2013			27.05783	142.21834	*Cinnamomum pseudopedunculatum*	Diploid
**KPN268**	2013			27.05530	142.21658	*Schima boninensis*	Diploid
**KPN270**	2013			27.05439	142.21645	*Rhaphiolepis indica* var. *umbellata*	Haploid
**KPN273**	2013			27.07182	142.21712	Broadleaf tree	Diploid
**KPN276**	2013			27.07217	142.21666	*Rhaphiolepis indica* var. *umbellata*	Diploid
**KPN278**	2013			27.07228	142.21649	*Mangifera indica*	Diploid
**KPN289** ^c^	2013			27.08087	142.21655	*Osmanthus insularis*	Diploid
**KPN299**	2013			27.09527	142.20975	*Rhaphiolepis indica* var. *umbellata*	Diploid
**KPN308**	2013			27.09522	142.20927	*Rhaphiolepis indica* var. *umbellata*	Diploid
**KPN332**	2013			27.09688	142.19466	*Rhaphiolepis indica* var. *umbellata*	Diploid
**KPN333**	2013			27.09688	142.19466	*Rhaphiolepis indica* var. *umbellata*	Diploid
**KPN257**	2012			27.05830	142.19478	*Morus australis*	Diploid
**KPN203**	2012		Haha-jima	26.65564	142.15241	*Rhaphiolepis indica* var. *umbellata*	Diploid
**KPN204**	2012			26.67795	142.14669	Broadleaf tree	Diploid
**KPN205**	2012			26.68171	142.14363	*Rhaphiolepis indica* var. *umbellata*	Diploid
**KPN206**	2012			26.69794	142.14303	*Morus australis*	Diploid
**KPN207**	2012			26.62729	142.17916	*Trema orientalis*	Diploid
**KPN309**	2013			26.62324	142.17892	*Planchonella obovata*	Diploid
**KPN212**	2012			26.62301	142.17853	*Rhaphiolepis indica* var. *umbellata*	Diploid
**KPN319**	2013			26.62433	142.17750	Broadleaf tree	Diploid
**KPN229**	2012			26.69555	142.14586	*Rhaphiolepis indica* var. *umbellata*	Diploid
**KPN231**	2012			26.64387	142.15549	*Leucaena leucocephala*	Diploid
**KPN233**	2012			26.64751	142.16940	*Ligustrum micranthum*	Diploid
**KPN238**	2012			26.65098	142.15992	*Cinnamomum pseudopedunculatum*	Diploid
**KPN246**	2012			26.65146	142.16913	Basidiocarp	Diploid
**KPN331**	2013			26.65146	142.16913	*Ficus elastica*	Diploid
**KPN318**	2013			26.62428	142.17763	*Celtis boninensis*	Diploid
**KPN321**	2013			26.67099	142.15536	Broadleaf tree	Diploid
**KPN323**	2013			26.67478	142.15578	Broadleaf tree	Diploid
**KPN328**	2013			26.70242	142.14421	Broadleaf tree	Diploid
**KPN330**	2013			26.70176	142.14467	Broadleaf tree	Diploid
**P919-02W.1** ^b^ ^**,**^ ^d^	2013	Federated States of Micronesia	Pohnpei	6.82381	158.17033	*Ficus tinctoria*	Diploid

^a^ Ploidy was determined from genotyping data.

^b^ Isolates used in first and second screening of microsatellite markers.

^c^ Isolates used in second screening of microsatellite markers.

^d^ Isolates used only in screening of microsatellite markers.

### DNA extraction

Fungal DNA was extracted from mycelia as described in Ota et al. (2014) [35]. Cultures were grown in 10 mL MYG medium (2% malt extract, 0.2% yeast extract, and 2% glucose) at 25°C in the dark and were harvested 7 days after inoculation. DNA was extracted from frozen mycelia using a DNeasy Plant Mini kit (Qiagen, Valencia, California) according to the manufacture’s instructions after grinding mycelia into a fine, dry powder using a mortar and pestle in liquid nitrogen.

### Microsatellite marker development

Genomic DNA (1 μg) extracted from *P*. *noxius* KPN92 were used to construct standard 350 bp libraries using the TruSeq DNA Sample Preparation Kit (Illumina, San Diego, California). Libraries were sequenced on an Illumina HiSeq2000 following the manufacturer’s recommended protocol to produce 100 bp paired-end reads. Assemblies of *P*. *noxious* genome sequences were constructed from Illumina reads using an MaSuRCA assembler [36] with the following options: GRAPH_KMER_SIZE = auto, ovlMerSize = 30, cgwErrorRate = 0.15, utgErrorRate = 0.015, and KMER_COUNT_THRESHOLD = 1.

MISA (http://pgrc.ipk-gatersleben.de/misa/) was used to identify di- to tri-nucleotide microsatellite loci from the genome assemblies of *P*. *noxius* KPN92 with at least eight repeats of di- and tri-nucleotides and the maximum number of bases between two microsatellite loci set to 100 bp. The total number of microsatellites identified was 334 (232 di-nucleotide microsatellite with 8–33 repeats and 102 tri-nucleotide microsatellite with 8–23 repeats). Specific primer pairs to amplify those microsatellite loci with four classes of product size were designed using Primer3 2.3.6 (http://primer3.sourceforge.net/) with the following options: the ranges of product size were 100–200, 200–300, 300–400, and 400–500. Within designated primer pairs, 50–60 pairs for each of the four product size classes (220 pairs in total) were selected arbitrarily and synthesised with the tail sequence on the 5’ end of the forward primer: tail A (GCC TCC CTC GCG CCA) for product size classes of 100–200 and 300–400 and tail B (GCC TTG CCA GCC CGC) for product size classes of 200–300 and 400–500 [37]. These 220 primer pairs were tested for amplification on eight *P*. *noxius* isolates: KPN1, 21, 92, 121, 145, 157, 256, and P919-02W.1 for the first screening. PCR amplification was performed using a BIO-RAD iCycler (Hercules, California) with the following conditions: 5 min at 95°C followed by 35 cycles of 30 s at 95°C, 30 s at 55°C, and 45 s at 72°C, and a final extension of 10 min at 72°C. Each PCR reaction contained approximately 5 ng template DNA, 0.2 μM of each primer, and 1X Go Taq Green Master Mix (Promega, Madison, Wisconsin) in 25 μL total volume. PCR products were separated by electrophoresis on a 1% agarose gel in TAE buffer and visualised using ethidium bromide staining on a UV transilluminator.

For the second screening, 20 primer pairs in each of the four product size classes (80 primer pairs in total) were arbitrarily selected from the primer pairs that generated clear PCR products in all eight isolates in the first screening; these were combined into 20 multiplex PCR panels that included one primer set of each of the four classes. To test the amplification of multiplex PCR panels and the polymorphism of each microsatellite loci, 15 *P*. *noxius* isolates were used: the 8 isolates used in the first screening, plus KPN28, 80, 132, 141, 280 and 289. PCR amplifications using a Qiagen Multiplex PCR Kit were performed with a BIO-RAD iCycler following the manufacturer’s recommended conditions: 15 min at 95°C followed by 35 cycles of 30 s at 94°C, 90 s at 55°C, and 60 s at 72°C, and a final extension of 30 min at 60°C. Each PCR reaction contained approximately 5 ng template DNA, 0.1 μM forward primer, 0.2 μM reverse primer, 0.2 mM of each universal primer labelled with fluorescent dye (Tail A with 6-FAM and Tail B with VIC, [37]), and 5 μL Master Mix (from the kit) in 10 μL total volume. Amplified products were loaded on an ABI 3130xl Genetic Analyzer (Applied Biosystems, Foster City, CA), and genotype scoring was performed using the GeneScan 600 LIZ dye size standard (Applied Biosystems) and GeneMapper version 4.1 software (Applied Biosystems). Finally, we selected 20 primer sets in five multiplex PCR panels for further analysis of the 128 Japanese isolates of *P*. *noxius*.

### Genetic diversity analysis

We defined isolates from the same island as a “population.” From the genotyping data, 102 isolates were observed as diploid, whereas 26 isolates were haploid. The number of alleles at each microsatellite locus was calculated using the program GenAlEx version 6.5.0.1 [38] for all 128 isolates. For further analysis of genetic diversity or genetic structure, haploid isolates were excluded. The number of alleles, Shannon’s information index (*I*), observed heterozygosity (Ho), expected heterozygosity (He), and Nei’s unbiased expected heterozygosity (uHe) for each locus were calculated using GenAlEx. Deviation from Hardy-Weinberg equilibrium (HWE) for each locus and linkage disequilibrium between loci were tested using the program Fstat version 2.9.3.2 [39] under the infinite allele model (IAM), and multiple testing with the Holm-Bonferroni method [40] was performed. Weir and Cockerham’s estimate of *F*_*IS*_ [41] was calculated using Fstat. These analyses were conducted among populations (each island) as well as between the two groups of populations (the Ryukyu Islands and the Ogasawara Islands) inferred from the STRUCTURE analysis.

### Genetic structure analysis

A Bayesian-based clustering method was applied to infer the genetic structure of Japanese *P*. *noxius* isolates using the program STRUCTURE version 2.3.4 [42]. An admixture model with correlated allele frequencies assuming no prior information of population origin was used. Twenty independent runs for *K* = 1 to 10 were performed at 100,000 Markov Chain Monte Carlo (MCMC) repetitions after a burn-in period of 50,000 iterations. The appropriate number of clusters (*K*) based on the *ad hoc* statics Δ*K* was determined using the method of Evanno et al. (2005) [43] with the program Structure Harvester [44].

Subsequently, analysis of molecular variance (AMOVA) was performed using the program Arlequin version 3.5.1.3 [45] to calculate the hierarchical distribution of genetic variation in Japanese isolates. All populations were initially combined into one hierarchical group, and then divided into two groups (the Ryukyu Islands and the Ogasawara islands) based on STRUCTURE analysis. The significance of components that showed variance was tested by performing 9,999 permutations.

Finally, the relationship between genetic structure and the isolation-by-distance (IBD) model was tested [46]. Values of *F*_*ST*_ between populations were calculated using Fstat. Mantel’s test was performed using GenAlEx with 9,999 permutations and assuming a linear relationship between pairwise values of *F*_*ST*_/(1-*F*_*ST*_) and the natural logarithm of geographic distance (km) between all population pairs [47]. The central value between the maximum and minimum latitude and longitude of the isolates on the same island was used as the location of the population.

## Results

### Characteristics of microsatellite markers

Of the 220 microsatellite primer pairs designed from the assembly of the *P*. *noxius* genome (isolate KPN92), 20 primer pairs in five multiplex panels were selected for use in the population analysis (Table 2). The 20 microsatellite markers were distributed in 19 distinct scaffolds in the genome assembly and exhibited high polymorphisms at each locus (Table 3). The sequences of these microsatellite loci inferred from the KPN92 genome assembly have been deposited in DDBJ (accession numbers are shown in Table 2). The number of alleles at each locus was 21.7 on average and ranged from 7 at Pn155 to 45 at Pn111 (Table 3).

**Table 2:** Characteristics of 20 microsatellite loci developed for *Phellinus noxius*.

Locus	Primer sequences (5' - 3')	Motif repeat	Tail label/ Multiplex panel^a^	Allele size range (bp)	Accession number
**Pn8**	F: TCGAGAACGAGGACGAGAGA	(AG)_15_	A/IV	191–258	LC064122
	R: ACCCTCTGCTTCTTCCTCCT				
**Pn11**	F: GGAGGGACACTGGGTAGGAA	(GAG)_10_	A/I	177–210	LC064123
	R: TCCCCTGTATGATCATCGGAGT				
**Pn14**	F: GAAAGGGGGAGACGGGAAAG	(GA)_9_	A/III	161–238	LC064124
	R: GGGGGAGTCGGTTTACATCC				
**Pn29**	F: TCTGTTTTACGTTGAGTCTCACA	(TCC)_8_	A/V	189–214	LC064125
	R: TGACAGCAATAAAGATAAGACGGG				
**Pn44**	F: TGCCAGTTTTGTAGTAGGCCT	(GAT)_13_	A/II	173–232	LC064126
	R: ACCACCTTGTCATTCGAGTGA				
**Pn71**	F: AGGCGGGCTTACTGATATGC	(TA)_9_	B/I	201–302	LC064127
	R: ACCCCTCGCAAATCCCAAAT				
**Pn78**	F: TTCCCCCTCCCCGAACTTAT	(ACT)_8_	B/III	272–304	LC064128
	R: CTTCGGACGACAAAGCTCCT				
**Pn83**	F: GCAACGAAGAAATGGCCTGG	(AG)_18_	B/IV	278–337	LC064129
	R: TATGTCCCGGCTTTGGCTTT				
**Pn84**	F: CTTGCTCTCCCGGAACCAAA	(GTT)_10_	B/V	267–293	LC064130
	R: CCAGGAGATCCGGGTATTAGA				
**Pn111**	F: AAAAACCTCGCCTACGGTGT	(GA)_19_	B/II	262–339	LC064131
	R: GGAGAAGAGACGTGAAGCCC				
**Pn131**	F: CTCAAGAACCCGAGGCTTGT	(AT)_12_	A/I	369–438	LC064132
	R: GTTCCGGACACAGTTCCCAT				
**Pn133**	F: GTCACGTGACTGCTATTACTTAGT	(TAT)_9_	A/III	323–357	LC064133
	R: CGGATCTTTTCTGTCACATTCCA				
**Pn140**	F: CGAGTTGGATCGGCTACTGG	(AAC)_9_	A/IV	279–387	LC064134
	R: GAGGGATGCGGTTAAGGCTT				
**Pn141**	F: CAGTCCCATCCGATACGAGC	(AT)_9_	A/V	368–408	LC064135
	R: TTCGCAAGCCAACGTTTCTG				
**Pn155**	F: TGGTGGTCAGGTTGAACGTC	(CAA)_9_	A/II	298–315	LC064136
	R: TATCGAAGCTTTCTGGCCGG				
**Pn175**	F: TCCCTCGTTCGTTTTTCCGT	(CT)_17_	B/IV	476–547	LC064137
	R: GGCTACTGAGAGTGGGGGTA				
**Pn178**	F: CCCTTCCTCACCCCACAAAA	(CT)_10_	B/I	505–547	LC064138
	R: GGGGCATGTTCTCACCTTCA				
**Pn210**	F: TTCGCGGTATGTTCAGCTCT	(CAT)_9_	B/III	405–465	LC064139
	R: CGCCTTTTTGTCGCAACTCA				
**Pn213**	F: AAAGAGGGCGTCTGGTTGTT	(TAA)_9_	B/V	488–525	LC064140
	R: TGGATTGTCATGGCGAGGTC				
**Pn214**	F: GTGGTAGTGGTAGTGGTGCC	(TGG)_8_	B/II	439–465	LC064141
	R: AACCTCCTTAACAAGCCCCG				

^a^ Sequence of the tail labels: A = GCC TCC CTC GCG CCA; B = GCC TTG CCA GCC CGC

**Table 3:** Summary of standard population genetics analysis for isolates in the Ryukyu Islands and the Ogasawara Islands.

	Total			Ryukyu (n = diploid 58 +haploid 25)						Ogasawara (n = diploid 44 +haploid 1)					
Locus	Na	Na^di^	He	Na	Na^di^	Ho	He	Rs	*F*_IS_ (W&C)	Na	Na^di^	Ho	He	Rs	*F*_IS_ (W&C)
**Pn8**	25	24	0.876	23	22	0.638	0.908	20.0	0.305*	5	5	0.591	0.716	5.0	0.186
**Pn11**	12	12	0.860	11	11	0.607	0.871	10.3	0.311	7	7	0.297	0.717	7.0	0.594*
**Pn14**	28	28	0.892	25	25	0.696	0.914	22.1	0.246*	7	7	0.568	0.722	6.8	0.224
**Pn29**	9	9	0.669	9	9	0.138	0.773	8.6	0.824*	1	1	0.000	0.000	1.0	–
**Pn44**	17	15	0.674	17	15	0.672	0.756	13.1	0.119	5	5	0.432	0.527	5.0	0.192
**Pn71**	29	29	0.904	27	26	0.776	0.890	21.8	0.136	12	12	0.750	0.846	11.3	0.124
**Pn78**	12	11	0.789	11	10	0.638	0.755	8.7	0.163	5	5	0.727	0.712	5.0	-0.010
**Pn83**	37	34	0.933	36	32	0.931	0.937	27.9	0.016	17	16	0.705	0.857	15.3	0.189*
**Pn84**	16	16	0.748	16	16	0.586	0.867	13.9	0.331*	4	4	0.227	0.394	3.8	0.433
**Pn111**	45	45	0.966	38	37	0.948	0.952	30.6	0.012	28	28	0.886	0.948	26.8	0.077
**Pn131**	25	25	0.877	22	21	0.776	0.910	18.9	0.156*	11	11	0.568	0.561	10.3	-0.001
**Pn133**	16	14	0.816	15	13	0.655	0.831	11.5	0.220*	6	6	0.591	0.768	5.8	0.242
**Pn140**	30	28	0.892	30	28	0.793	0.892	23.0	0.120	6	6	0.750	0.799	6.0	0.073
**Pn141**	19	18	0.841	17	15	0.741	0.852	13.0	0.138	8	8	0.545	0.718	7.5	0.251
**Pn155**	7	7	0.585	7	7	0.362	0.360	6.1	0.004	2	2	0.023	0.022	1.8	0.000
**Pn175**	36	33	0.942	36	32	0.397	0.955	27.8	0.590*	14	14	0.409	0.885	13.6	0.546*
**Pn178**	13	13	0.752	12	12	0.552	0.629	10.3	0.131	5	5	0.386	0.465	4.8	0.180
**Pn210**	33	31	0.889	29	27	0.810	0.924	23.3	0.132	10	10	0.545	0.696	9.3	0.227
**Pn213**	15	14	0.723	14	13	0.552	0.845	11.5	0.355*	5	5	0.318	0.334	4.9	0.059
**Pn214**	10	9	0.809	10	9	0.690	0.825	8.5	0.172	7	7	0.568	0.760	6.7	0.263
**All Loci**	21.7	20.8	0.822	20.3	19.0	0.648	0.832	16.5	0.224*	8.3	8.2	0.495	0.623	7.9	0.217*
**SE**	2.4	2.3	0.023	2.2	2.0	0.043	0.031	1.7	0.044	1.4	1.3	0.053	0.059	1.3	0.040

Na, Na^di^, Ho, He, and Rs refer to as the total number of alleles per locus in all isolates, the total number of alleles per locus in diploid isolates, the observed heterozygosity, the expected heterozygosity, and allelic richness respectively. *F*_IS_ was calculated by Weir & Cockerham.

* indicates that the HWE test is significant after the Holm-Bonferroni correction method (α = 0.05).

–indicates that *F*is was not calculated because the loci was monomorphic.

In all, 4 to 22 isolates of *P*. *noxius* from each island were tested using 20 microsatellite markers, and remarkably, all 128 isolates exhibited different genotypes. A total of 102 multilocus genotypes were interpreted as being diploid from the microsatellite analysis; however, 25 of 83 isolates from the Ryukyu Islands and 1 of 45 isolates from the Ogasawara Islands were judged to be haploid, because one single allele was detected at all loci in these isolates. Only diploid isolates were used for further analysis of genetic structure and diversity. In addition, the Yoron Island population was excluded because all of its isolates were haploid.

### Genetic diversity

A summary of the genetic diversity for the 14 populations is presented in Table 4. Expected heterozygosity across all populations was 0.67 (±0.01 SD), ranging from 0.48 (±0.07) on Okinoerabu Island to 0.81 (±0.03) on Miyako Island. Unbiased expected heterozygosity exhibited the same trend as expected heterozygosity. Shannon’s diversity index (*I*) was 0.81–1.95 (mean = 1.46) for the 11 populations in the Ryukyu Islands and 0.96–1.30 (mean = 1.18) for the three populations in the Ogasawara Islands. There was a low number of isolates for some populations; therefore, for further analysis, each population was combined into two groups of islands (the Ryukyu Islands and the Ogasawara Islands) based on the STRUCTURE analysis. For the Ryukyu Islands (N = 58), the average observed and expected heterozygosity at each locus was 0.648 ± 0.043 and 0.832 ± 0.031, respectively. *F*_IS_ at each locus ranged from 0.004 to 0.824, and significant deviation from HWE was detected at 9 of 20 loci after sequential Bonferroni correlation (α = 0.05). For the Ogasawara Islands (N = 44), the average observed and expected heterozygosity at each locus was 0.495 ± 0.053 and 0.623 ± 0.059, respectively. *F*_IS_ at each locus ranged from -0.001 to 0.594, and significant deviation from HWE was detected at three loci after sequential Bonferroni correlation (α = 0.05). Allelic richness was 16.5 ± 1.7 in the Ryukyu Islands and 7.9 ± 1.3 in the Ogasawara Islands. No significant linkage disequilibrium was detected between each locus in any population after sequential Bonferroni correlation (α = 0.05).

**Table 4:** Genetic diversity across 16 populations (islands) of *Phellinus noxius*.

Island	N	N^di^	*I*	Ho	He	uHe
**Ryukyu Islands**						
**Amami-Oshima**	8	5	1.49 ± 0.10	0.66 ± 0.07	0.71 ± 0.04	0.79 ± 0.05
**Kikai**	7	4	1.40 ± 0.08	0.70 ± 0.07	0.70 ± 0.03	0.80 ± 0.03
**Tokunoshima**	6	3	1.04 ± 0.10	0.48 ± 0.07	0.58 ± 0.05	0.70 ± 0.05
**Okinoerabu**	4	2	0.81 ± 0.12	0.58 ± 0.10	0.48 ± 0.07	0.63 ± 0.09
**Yoron**	6	0				
**Okinawa**	11	8	1.82 ± 0.10	0.67 ± 0.05	0.79 ± 0.02	0.84 ± 0.03
**Iheya**	5	5	1.50 ± 0.11	0.69 ± 0.06	0.70 ± 0.04	0.78 ± 0.04
**Kume**	6	5	1.49 ± 0.09	0.70 ± 0.07	0.73 ± 0.03	0.81 ± 0.03
**Tokashiki**	4	4	1.42 ± 0.11	0.70 ± 0.06	0.70 ± 0.04	0.80 ± 0.04
**Miyako**	13	11	1.95 ± 0.1	0.58 ± 0.04	0.81 ± 0.03	0.84 ± 0.03
**Ishigaki**	4	3	1.43 ± 0.05	0.78 ± 0.04	0.73 ± 0.02	0.88 ± 0.02
**Iriomote**	9	8	1.71 ± 0.11	0.63 ± 0.06	0.76 ± 0.03	0.81 ± 0.04
**Ogasawara Islands**						
**Ani-jima**	4	4	0.96 ± 0.14	0.53 ± 0.08	0.50 ± 0.07	0.57 ± 0.08
**Chichi-jima**	22	21	1.30 ± 0.16	0.53 ± 0.06	0.59 ± 0.06	0.61 ± 0.06
**Haha-jima**	19	19	1.27 ± 0.17	0.44 ± 0.05	0.61 ± 0.06	0.63 ± 0.06
**Total**	128	102	1.40 ± 0.03	0.62 ± 0.02	0.67 ± 0.01	0.75 ± 0.01

N, N^di^, *I*, Ho, He, and uHe refer to as number of all isolates, number of diploid isolates, Shannon's Information index, observed heterozygosity, expected heterozygosity, unbiased expected heterozygosity, respectively.

### Genetic structure

**Fig 2:**
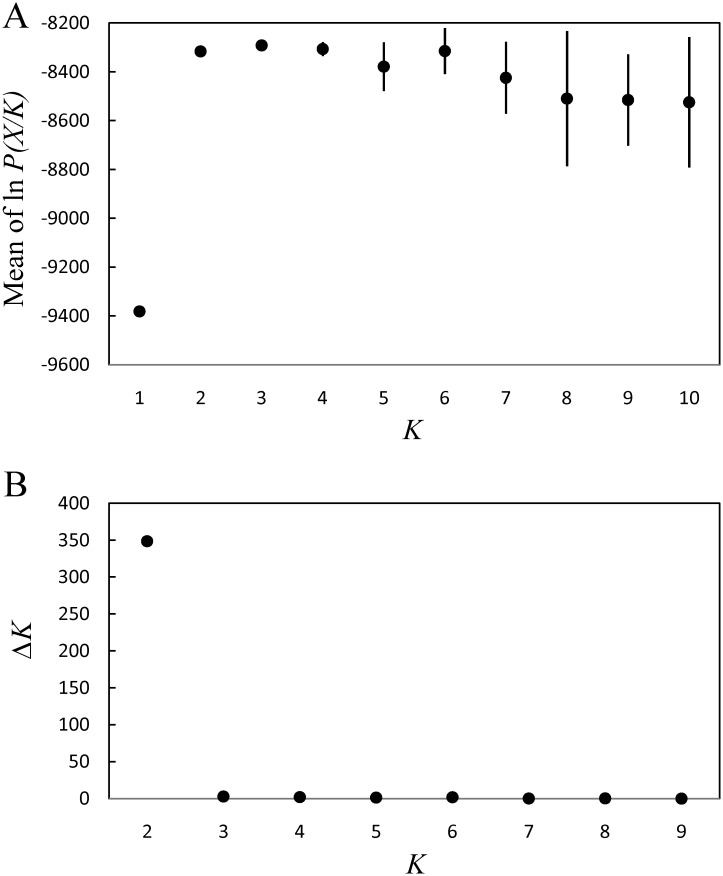
A) Values of log likelihood of the data, ln *P*(*X*/*K*), as a function of the number of clusters, K, from STRUCTURE analysis. B) Value of Δ*K*, based on the rate of change in ln *P*(*X*/*K*) between successive *K* values generated from Structure Harvester. Each bar indicates the standard deviation of 20 independent runs.

**Fig 3:**
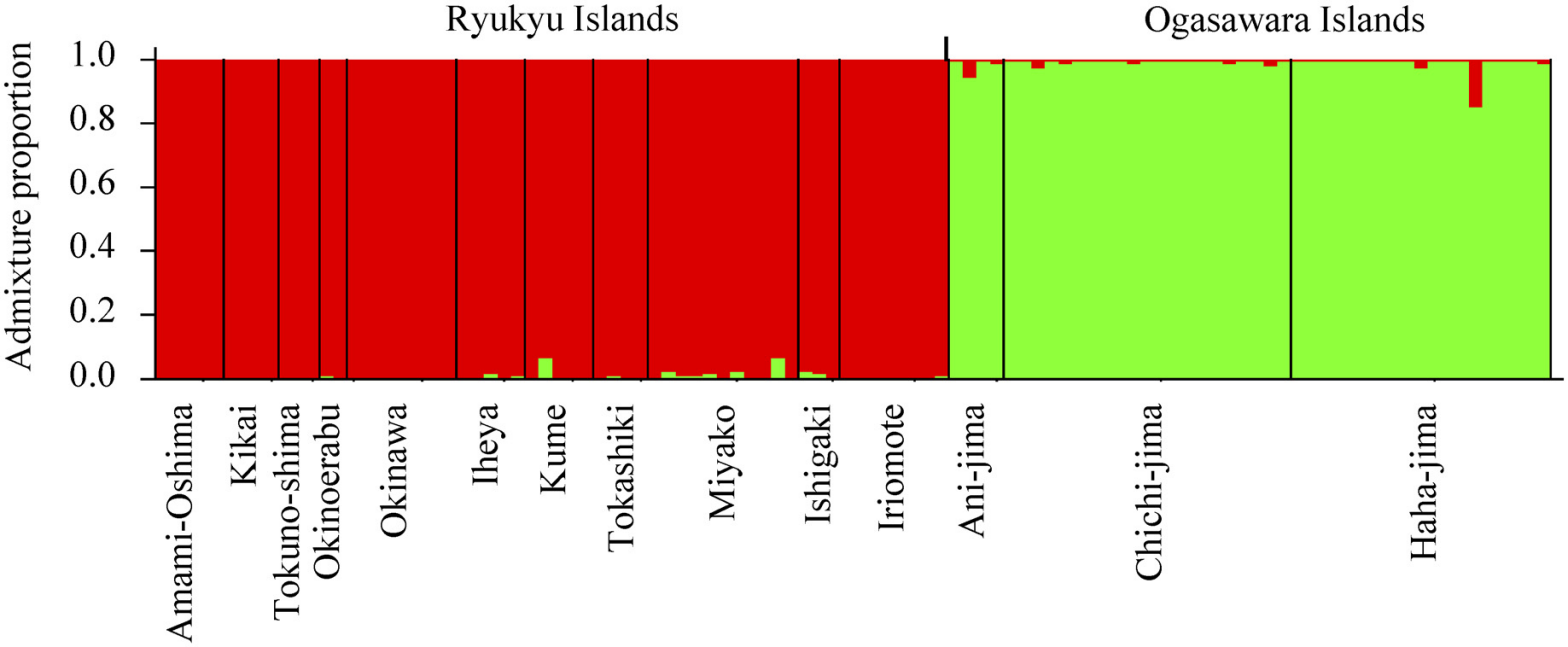
Bar plots of the coefficients of co-ancestry obtained from STRUCTURE analysis with *K* = 2. Each bar corresponds to one individual isolate, and each cluster is represented by a particular colour.

Evanno’s method using Structure Harvester clearly indicated that Δ*K* at *K* = 2 was at a maximum and two was an appropriate number of clusters (Fig 2). The two clusters clearly exhibited structure between isolates of the Ryukyu Islands (composed of 11 islands from Amami-Oshima Island to Iriomote Island) and those of the Ogasawara Islands, containing the islands of Ani-jima, Chichi-jima, and Haha-jima (Fig 3).

When all populations were combined in one hierarchical group, AMOVA analysis indicated that most of the genetic variation could be explained by differences in individual isolates within populations (85.52%) rather than by variation among populations (14.48%, *P* < 0.0001, Table 5). When the populations were partitioned into two groups (the Ryukyu Islands and the Ogasawara Islands) established from the STRUCTURE analysis, most of the genetic variance could be explained by differences in individual isolates within a population (79.18%, *P* < 0.0001). Differences in isolates among groups and among populations within groups explained 16.65% (*P* = 0.0029) and 4.17% (*P* < 0.0001), respectively.

**Table 5:** Analysis of molecular variance (AMOVA) for *Phellinus noxius* populations using 20 microsatellite loci.

Source of variation	*df*	sum of squares	variance component		% of variation	*P* value
**Among population**	13	295.96	1.15	Va	14.48	<0.0001
**Within population**	190	1290.74	6.79	Vb	85.52	
**Total**	203	1586.70	7.94			
*F*_ST_ = 0.14478						
**Among groups**	1	159.36	1.43	Va	16.65	0.0029
**Among population within groups**	12	136.60	0.36	Vb	4.17	<0.0001
**Within population**	190	1290.74	6.79	Vc	79.18	<0.0001
**Total**	203	1586.70	8.58			
*F*_SC_ = 0.05008, *F*_ST_ = 0.20825, *F*_CT_ = 0.16651						

The analysis included all diploid isolates as one hierarchical group, and partitioning populations into two groups (the Ryukyu Islands and the Ogasawara Islands) inferred from STRUCTURE analysis.

**Fig 4:**
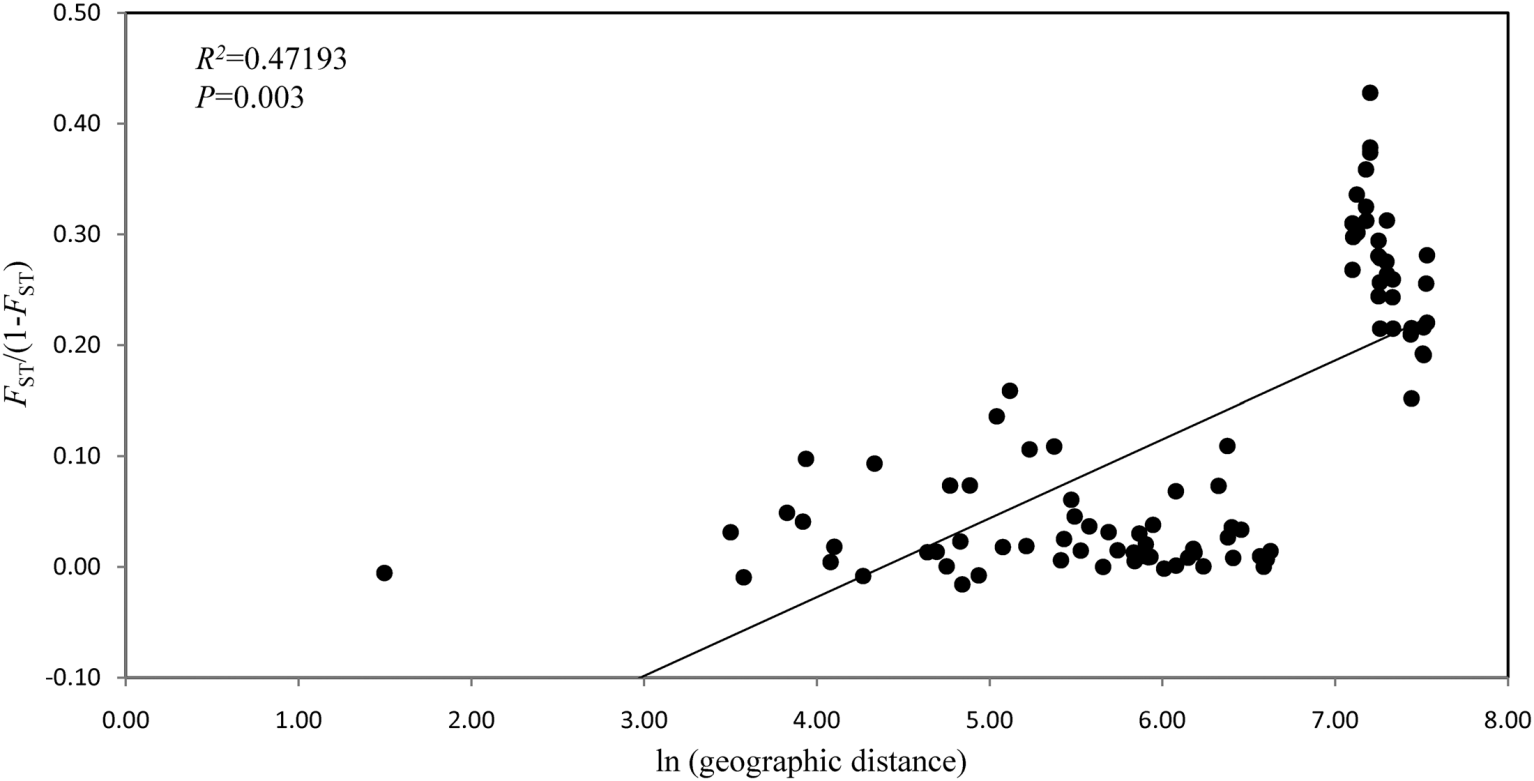
Relationship between the pairwise genetic distance, *F*_*ST*_/(1-*F*_*ST*_), and geographic distance among 14 populations (islands).

A pairwise analysis of IBD among populations indicated a significant positive correlation between genetic distance and geographic distance (*R* ^2^ = 0.47193; *P* = 0.003, Fig 4). High *F*_*ST*_/(1-*F*_*ST*_) values above 1200 km (= ln7.1 km) were consistent with the pairwise analysis between the Ryukyu Islands and the Ogasawara Islands.

## Discussion

We developed 20 microsatellite markers for *P*. *noxius*. Multiplex PCR for these markers (four of each primer pair in one reaction) successfully genotyped Japanese isolates as well as an isolate from Pohnpei Island, Federated States of Micronesia, indicating that these markers are useful to genotype isolates from other geographic regions. Moreover, these markers showed enough polymorphism to analyse the genetic or clone composition of *P*. *noxius* in local populations. For some Ryukyus Islands, we were unable to obtain a sufficient number of isolates to analyse differences between islands, because the number of sites where the disease occurred was too low and some isolates were haploid. However, the number of isolates was sufficient for comparisons between the Ryukyu Islands and the Ogasawara Islands.

Diploidy is the main ploidy for vegetative hyphae in Hymenochaetaceae, which is consistent with our microsatellite data for *P*. *noxius*. However, 26 of 128 isolates obtained from decayed woods or basidiocarps were judged to be haploid. Among the isolates from the Ryukyu Islands obtained between 1999 and 2014, 30.1% were haploid, whereas only one isolate was haploid among those isolated from the Ogasawara Islands during or after 2012 (Table 1). During the maintenance of the isolate cultures, we encountered cases in which some sub-cultures from a diploid isolate showed haploid microsatellite signals that had only one of two alleles of each microsatellite loci of diploid isolates. In *Pholiota nameko*, an edible basidiomycetous fungus, diploid mycelia often become haploid during storage via a mechanism known as monokaryotisation or dedikaryotisation [48,49]. Haploid mycelia of *P*. *noxius* may occur as primary mycelia that are derived from germinated basidiospores, however, basidiocarp formation of this fungus is very rare [3,12] and the primary mycelia are usually short lived [50]. Therefore, haploid *P*. *noxius* isolates in this study may have changed from diploid during periodical subculturing by monokaryotisation.

*Phellinus noxius* has two dissemination methods: asexual root-to-root contact from a diseased tree to a living tree and dispersal of sexually produced basidiospores [1,14]. Using somatic incompatibility tests, Hattori et al. [51] examined the clone distribution of *P*. *noxius* in windbreak trees on the Ishigaki Islands of Japan. They concluded that infection via both basidiospores and root-to-root contact occurred in the area. Some researchers, however, have suspected that infection by basidiospores is rare because basidiocarps are seldom seen in areas of disease propagation [3,12]. We found that all of the isolates exhibited unique genotypes, strongly indicating that basidiospore infection is the main dissemination method for the formation of new disease foci. In the Ryukyu Islands, basidiocarps were rarely seen in areas where the disease was spreading and forming forest gaps [14]; however, they were occasionally seen on dead or fallen trees in natural forests, where the disease was not spreading. Meanwhile, basidiocarps are more frequently observed on the Ogasawara Islands, although the reason is unclear (Hattori personal observation). In such areas, basidiospores may function to produce new disease foci. Although many unique genotypes have been observed, it is possible that a small number of genotypes dominates within a single disease focus, because *P*. *noxius* spreads asexually within disease foci like *Phellinus sulphurascens* and *Armillaria* spp [1,15,17,18]. More extensive sampling within disease foci are needed to clarify the clone distribution pattern of *P*. *noxius*.

Whether *P*. *noxius* is indigenous in Japan or introduced from other areas is unknown. Because brown root rot was first recognised in Japan as recently as the 1980s, the possibility of *P*. *noxius* as an introduced pathogen has been expected [23]. Ann et al. [12] suggested that *P*. *noxius* was likely introduced to Taiwan on diseased roots of exotic trees, based on observations that the distribution of *P*. *noxius* in Taiwan is limited to areas of human activity and the disease has never been found in undisturbed forests. In general, introduced pathogens have lower genetic diversity than indigenous pathogens because of the founder effect of small population sizes and subsequent bottlenecks [52]. Introduced diseases that have had devastating effects include chestnut blight caused by an ascomycetous fungus *Cryphonectria parasitica* (Murrill) M.E. Barr [27], ash dieback by *Hymenoscyphus fraxineus* (T. Kowalski) Baral, Queloz & Hosoya [53], sudden oak death by an oomycete *Phytophthora ramorum* Werres, De Cock & Man in't Veld [54], and alder decline due to *P*. *alni* Brasier & S.A. Kirk [28]. Population genetics studies using microsatellite markers have indicated that the genetic diversities of these species in the area of introduction are lower than in native areas [27,28,53]. In terms of root-rotting basidiomycetous fungi, *Heterobasidion irregulare* Garbel. & Otrosina in Italy [29] and *Armillaria mellea* (Vahl) P. Kumm in South Africa [55] are known as introduced pathogens. *Heterobasidion irregulare* in Italy, which is suspected to have been introduced by the US military during World War II, exhibits fewer alleles (1–7) at each microsatellite locus than native populations in North America. In our study, Japanese *P*. *noxius* isolates exhibited a high number of alleles per loci (21.7 on average), suggesting that *P*. *noxius* is indigenous to Japan or was introduced to the country a very long time ago. Further studies using isolates collected from other geographic region are needed to confirm the conclusion. Although the occurrence of brown root rot in Japan was only recognised recently (i.e., in the 1980s on the Ryukyu Islands and the 2010s on the Ogasawara Islands), basidiocarps of *P*. *noxius* were recorded on a broadleaved tree on the Ogasawara Islands in 1916 [56]. This suggests that *P*. *noxius* was present on these islands without causing a conspicuous decline of resident trees. The causes of the recent outbreak of this disease in Japan has not yet been determined, although several environmental changes, including irregular climatic events such as typhoons and droughts, as well as human disturbances may have contributed to the outbreak.

The STRUCTURE analysis strongly indicated genetic differentiation between the Ryukyu and Ogasawara populations of *P*. *noxius*. Additionally, the AMOVA and IBD analysis also supported the conclusion. These findings suggest minimal gene flow between the two island chains over a long period of time or a different origin of the two populations. The Ryukyu Islands and Taiwan are continental islands that were once connected to the Eurasian continent; thus, *P*. *noxius* was able to spread to and from the continent similar to other flora and fauna [57]. In contrast, the Ogasawara Islands are oceanic islands formed by volcanic activity and were never connected to a continent or other larger islands such as the main Japanese islands. Therefore, the origins of all flora and fauna on them are thought to be introductions from other continents or islands followed by their unique evolution. Because the dispersal modes for plants on the Ogasawara Islands are by air, bird, and oceanic drift [57], *P*. *noxius* was probably introduced via one of these methods. In general, basidiospores are ephemeral, and the majority of basidiospores fall within a short distance of the basidiocarps [58,59]. However, long-distance dispersal (1000 km) has also been reported in some wood-inhabiting basidiomycetous fungi [60]. The basidiospores might have been introduced from the Mariana Islands, the nearest oceanic islands to the Ogasawara Islands, by a typhoon, as many typhoons form around the Marianas and move to the Ogasawaras.

*Phellinus noxius* could serve as a suitable model for studying the evolutionary history of fungi and forest diseases on oceanic islands, because it is distributed in three geographically different categories: continents, continental islands, and oceanic islands. Further population genetics studies using isolates collected from around the world will be useful for understanding the evolutional history of *P*. *noxius* and its worldwide routes of dispersal.
